# Morpho-Chemical Observations of Human Deciduous Teeth Enamel in Response to Biomimetic Toothpastes Treatment

**DOI:** 10.3390/ma13081803

**Published:** 2020-04-11

**Authors:** Maurizio Bossù, Roberto Matassa, Michela Relucenti, Flavia Iaculli, Alessandro Salucci, Gianni Di Giorgio, Giuseppe Familiari, Antonella Polimeni, Stefano Di Carlo

**Affiliations:** 1 Department of Oral and Maxillofacial Science, Sapienza University of Rome, Via A. Borelli 50, 00161 Rome, Italy; maurizio.bossu@uniroma1.it (M.B.); flavia.iaculli@uniroma1.it (F.I.); alessandro.salucci@uniroma1.it (A.S.); gianni.digiorgio@uniroma1.it (G.D.G.); antonella.polimeni@uniroma1.it (A.P.); stefano.dicarlo@uniroma1.it (S.D.C.); 2 Department of Anatomical, Histological, Forensic and Orthopaedic Sciences, Section of Human Anatomy, Sapienza University of Rome, Via A. Borelli 50, 00161 Rome, Italy; michela.relucenti@uniroma1.it (M.R.); giuseppe.familiari@uniroma1.it (G.F.)

**Keywords:** biomaterials, toothpaste, enamel, deciduous, diffusion, hydrate, electron microscopy, microanalysis, EDS, mapping, scanning electron microscopy

## Abstract

Today, biomaterial research on biomimetic mineralization strategies represents a new challenge in the prevention and cure of enamel mineral loss on delicate deciduous teeth. Distinctive assumptions about the origin, the growth, and the functionalization on the biomimetic materials have been recently proposed by scientific research studies in evaluating the different clinical aspects of treating the deciduous tooth. Therefore, appropriate morpho-chemical observations on delivering specific biomaterials to enamel teeth is the most important factor for controlling biomineralization processes. Detailed morpho-chemical investigations of the treated enamel layer using three commercial toothpastes (Biorepair, F1400, and F500) were performed through variable pressure scanning electron microscopy (VP-SEM) and energy dispersive X-ray spectroscopy (EDS) on deciduous teeth in their native state. A new microscopy methodology allowed us to determine the behaviors of silicate, phosphate, and calcium contents from the early stage, as commercially available toothpastes, to the final stage of delivered diffusion, occurring within the enamel layer together with their penetration depth properties. The reported results represent a valuable background towards full comprehension of the role of organic–inorganic biomaterials for developing a controlled biomimetic toothpaste in biofluid media.

## 1. Introduction

Human teeth are constantly subjected to a continuous process of de- and re-mineralization [1]. The imbalance of this process due to cariogenic bacteria, external agent, heat, cold, erosive food and drink causes loss of mineralized tooth tissues as well as dental decay. This is mostly true for primary teeth, since the enamel of deciduous teeth appears to be thinner than the permanent ones, and lesser mineralized (mineralization of 80.6% in deciduous enamel and 89.7% in permanent enameled teeth) with not completely formed prisms at the outer layer, called aprismatic enamel [2]. Whittaker et al. [3] reported that 60% of deciduous teeth showed an aprismatic surface zone of 16–45 μm in thickness, while 50% of the permanent teeth was estimated as having an aprismatic layer less than 5 μm. As it is not a living tissue, enamel is not able to self-repair thereby it loses mineral due to chemical and mechanical external agents. 

In order to prevent teeth demineralization and promote re-mineralization, several biomaterials have been used to aim at restoring enamel and dentine tissues. Particularly, the ability of fluorine to re-mineralize dental surfaces has been widely demonstrated for both permanent and deciduous teeth [1]. Fluoride-containing toothpastes, or other fluoride supplements, have been successfully introduced as a global solution to mostly prevent dental caries and promote tooth re-mineralization [4]. However, the potential risk of fluorosis should be accurately balanced with the caries-preventive effect of fluorine and a greater attention has to be paid to fluoride toothpaste concentrations as well as the systemic supply. This is mostly true in children of less than 5 years of age, due to the swallowing of a high amount of fluoride toothpaste during the mineralization phase of permanent teeth [5]. Therefore, dental material research should be addressed to develop biomimetic materials that have performance in terms of prevention and remineralization. 

Among the employed biomaterials commercially available, toothpastes are the mainly used agents in preventive dentistry. The graininess in terms of dimension and chemical composition can be modulated for delivering the appropriate diffusion of adequate chemical constituents throughout the extraordinary enamel microarchitecture. Thus, expanding the study of enamel deciduous teeth with the design flexibility of biomaterial toothpaste of both morphological and chemical features, remains a considerable challenge. Therefore, the magnesium (Mg), phosphorous (P), and calcium (Ca), basic chemical elements of natural teeth, need to be tailored to enrich the enamel mineralization processes. In particular, the morpho-chemical distribution of the enamel deciduous layer and its biomimetic mineralization, using toothpaste at the microscopic scale has not yet been observed properly. In fact, the current development of toothpaste and gel approaches for teeth enamel require appropriate characterization to be able to provide morpho-chemical details to understand the fundamental delivery diffusion of the chemical constituents through the enamel architectures. To the best of our knowledge, only a few studies have so far explored the chemical diffusion effect of toothpaste on adult teeth enamel. Guentesch et al. [6] reported the penetration depth as a function of the morpho-chemical contents on enamel and dentin adult teeth by VP-SEM and which were chemically analysed by line scan EDS profile techniques. Furthermore, the diffusion effect of new composite material through demineralized human dentin was analyzed by line scan EDS on rough surfaces [7]. On the other hand, other biomimetic mineralization studies on pathological permanent dental features were only focused on calculating the Ca/P ratio (EDS technique) on an average extensive micrometric area for determining the regeneration crystal of enamel caries-like lesion by arginine (Arg) in sodium fluoride (NaF) toothpaste [8]. Highly concentrated stannous fluoride (SnF_2_) and NaF solutions and a NaF/CaF_2_ varnish on adult treated enamel have been analyzed in terms of erosion and abrasion by scanning electron microscopy (surface morphology) and EDS microanalysis [9]. Shen et al. compared remineralization of adult enamel subsurface lesions treated with dental products in excess of calcium phosphate by means of densitometry profile, microradiography, and ion chromatography [10]. Since deciduous and permanent enamel teeth differ in chemical, morphological, and physiological aspects, this implies different behavior under physiological conditions such as the erosion process and caries. Due to this, the response of deciduous teeth enamel to biomimetic toothpaste treatments may be of central interest in terms of potential caries prevention. Therefore, the morpho-chemical investigations of commercial toothpastes have been focused on their ability to diffuse into the deciduous enamel layer to investigate the protective role and the dose- dependent modulation to remineralize the crystal habitat. To obtain insight into the diffusion of these biomaterials at the microscopic scale, a combination of microscopy (VP-SEM, EDS spectrum and mapping), and image processing techniques have been exploited in order to obtain all possible experimental information to explore the following objectives: (*i*) to conduct a morpho-chemical analysis of deciduous teeth treated by commercially available toothpastes; (*ii*) to propose a new microscopy methodology of diffusion characterization that would be useful for investigating the performance of commercial dental products in terms of chemical graininess synthesis; (*iii*) to evaluate the ability of biomimetic toothpastes to diffuse into the deciduous enamel layer to protect remineralization of the crystal habitat. These aspects not only would improve the synthesis of novel biomaterials specifically designed for deciduous teeth, but also would represent a positive global economic impact because of the wide prevalence of dental caries affecting primary teeth [1].

## 2. Materials and Methods

### 2.1. Samples Preparation

In the present study three different toothpastes were compared: (a) commercial toothpaste containing fluorine 500 ppm (Elmex® bimbi, Colgate-Palmolive S.p.a, Roma, Italy); (b) commercial toothpaste containing fluorine 1400 ppm (Elmex® Junior, Colgate-Palmolive S.p.a, Roma, Italy); (c) toothpaste containing hydroxyapatite nanocrystal (Biorepair®, Coswell S.p.a., Funo, Bologna, Italy). To evaluate the ability of toothpastes to diffuse into the deciduous enamel layer, 8 primary teeth were extracted as a result of orthodontic treatments or physiological replacements. The procedures were performed at the Pediatric Dentistry Unit of “Sapienza” University of Rome. Parents of all subjects gave their informed consent during the first visit to the Pediatric Dentistry Unit allowing the use of extracted teeth for research purposes. The study was conducted in accordance with the Declaration of Helsinki, and the protocol was approved by the Department Committee of the Department of Oral and Maxillofacial Science, Sapienza University of Rome (No. 16/2020 Prot. No. 0000427). The teeth were processed as previously described by Bossù et al. [11]. Briefly, once extracted, each element was preserved in normal saline solution and then divided at a cementum-enamel junction level, by means of diamond-tipped saw Secotron 200, in order to remove the root portion. Subsequently, the specimens were fixed on composite resin supports and subjected to etching with 37% orthophosphoric acid for 1 min to reproduce the physiological demineralization that occurs in the oral environment, and then rinsed with normal saline solution. Two teeth per group were manually brushed for 15 days, three times a day, using 3 different pediatric toothbrushes (one for each group) for 2 min, as follows: (a) commercial toothpaste containing fluorine 500 ppm; (b) commercial toothpaste containing fluorine 1400 ppm; (c) toothpaste containing hydroxyapatite nanocrystal (Biorepair). The elements used as control (n = 2) were subjected to the same manual brushing, with an additional toothbrush, although with water, 3 times a day for 15 days. After every treatment, the samples were rinsed and preserved at room temperature in normal saline solution, which was renewed at every brushing session. 

### 2.2. Variable Pressure Scanning Electron Microscopy

Hydrated toothpastes and deciduous teeth were observed using a variable pressure scanning electron microscopy (VP-SEM, Hitachi SU-3500) with dual energy dispersive X-ray spectroscopy detectors (VP-SEM-dEDS) arranged in parallel configuration (Bruker, XFlash®660). Samples, prepared as described in Section 2.1, were cross-sectioned from the apical side to avoid artifacts producing processes that could compromise the morphology and EDS microanalysis investigations [12,13,14]. The samples were directly settled onto a carbon planchet stub without conductive coating and the naturally obtained cross-sectioned samples were not flattened and polished. A thin film of distilled water was deposited onto the hydrated samples at room temperature to limit water-loss during low vacuum procedures. By appropriate control of the chamber pressure and cooling temperature, particular attention was paid to avoid crack formations of the enamel structures. All samples were observed at an accelerating voltage depending on the features of the correlative pressure/temperature used in the chamber to avoid radiation damage and drifting image, fatal for EDS multi-mapping analyses.

## 3. Results

**Figure 1:**
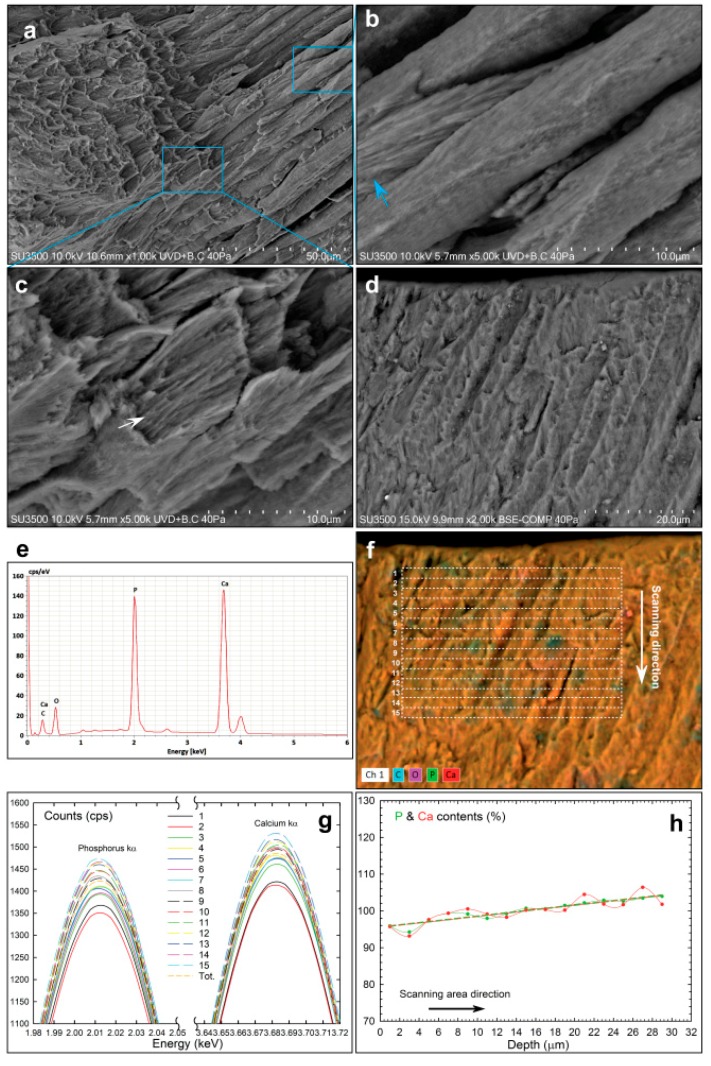
Morpho-chemical study of untreated deciduous enamel tooth. (**a**) VP-SEM image illustrating the inner surface morphology. (**b**) VP-SEM magnification showing crystalline rods wrapped in a sheath of enamel matrix (blue arrow). (**c**) VP-SEM magnification showing aligned fracture rods (white arrow). (**d**) VP-SEM image of cross-sectioned enamel close to the outer surface (**e**) EDS spectrum probed in Figure 1d. (**f**) Elemental mapping image of major constituents overlapped with a cross-pattern denoted by a white dotted line rectangle. (**g**) Plots showing the characteristic peak energy of P-kα and Ca-kα corresponding to each rectangular area of the Figure 1f. (**h**) Experimental plot showing atomic percentage distribution of P and Ca elements along the scanning area direction (dotted line) and data fitted by linear regression (short dashed line).

The cross-section of the deciduous tooth shows the typical microstructures of discontinuous and fragmented enamel crystals observed in their native state by VP-SEM at about the middle enamel layer (Figure 1a).

The hydrated enamel fracture exhibits well-packed crystalline rods relatively close and almost parallel with minimal interprismatic matrix area. The enamel rods of a deciduous enamel have an average size of 4.63 ± 0.12 μm slightly less than an adult enamel [15]. Each rod is wrapped in a sheath of enamel matrix with an interspacing rod of 1.05 ± 0.31 μm on average (Figure 1b, blue arrow), which interacts with the enamel rod crystal to guide and participate in the cooperative self-assembly of the high-order crystalline growth. Inside the rods are evidently aligned and fractured nanometric bundles that may range from 350 to 450 nm in size (Figure 1c, white arrow). These enamel hierarchical structures in nature are a cooperative interaction between matrix assembly and well-organized apatite crystal growth [16]. The EDS spectrum in Figure 1e of untreated deciduous was probed on the outer cross-sectioned enamel of Figure 1d. The major concentration of Ca and P with minor concentrations of C, O, Mg and trace amounts of Na and Cl (technical back-ground) were evidence of formed hydroxyapatite, providing a Ca/P molar ratio of 1.54 ± 0.39% [15]. Multi-elemental dual-EDS mapping of O, C, P, Ca elements seems uniformly distributed on the entire cross-section enamel (Figure 1f). To obtain insight into the multi-elemental spatial distribution, different spectra were taken from a selected cross-pattern of the smallest micrometric area, each with a count time of up to 65 Ks. By properly analysing the change of the P and Ca contents along the scanning depth of the enamel layer, the EDS spectra were collected within each rectangular area of 2 × 40 μm^2^ from the outer to the inner enamel layer sequentially (labelled from 1 to 15), forming a microarray of 30 × 40 μm^2^ (white dashed lines of Figure 1f). It should be noted that the obtained surface by natural cutting action was not sufficiently smooth to perform a quantitative EDS line scan profile since this microanalysis technique is strictly dependent on the surface roughness. Apparently, the overlapping image maps evidenced a heterogeneous spatial distribution of the C, O, P and Ca elements in the field of view of the cross-section. The different intensity colour in the smallest micrometric regions might be related to the formation of crystalline hydroxyapatite in micro domains. The changing of the P and Ca amounts along the enamel crystal rods partially covered by the interprismatic matrix is clearly shown by the variation in amplitude and in width of the corresponding energy peaks (Figure 1g). For quantifying the P and Ca contents detected in each EDS frame map, each K_α_ peak was processed by Gaussian fitting to estimate the corresponding peak area [17]. The estimated areas of each characteristic P and Ca peaks were normalized by the corresponding peak area belonging to the entire probed area of 30 × 40 μm^2^. This action was useful for light element analysis and for the detected atomic percentage, relatively insensitive to small changes in surface geometry. The ratio of the total integrated counts quantitatively provided the atomic percentage of P and Ca elements between the different spectra acquired along the scanning depth from the outer to the inner surface of the enamel layer (Figure 1h). However, it should be noted that the ratio of the P and Ca peak area exhibited an oscillating difference along the scanning depth profile in which the percentages of phosphate were similar or slightly great than calcium content. To gain a deeper insight into the atomic percentage distributions across the enamel layer, the P and Ca experimental functions were both fitted by linear regression providing intercept values of 95 % and positive slope of 0.30 (Table 1), assumed as reference for the treated deciduous teeth. These results support a low crystallinity organization and an amount of P and Ca closer to the outer enamel surface progressively increasing along the scanning area direction of the growing crystal apatite habitat.

**Figure 2:**
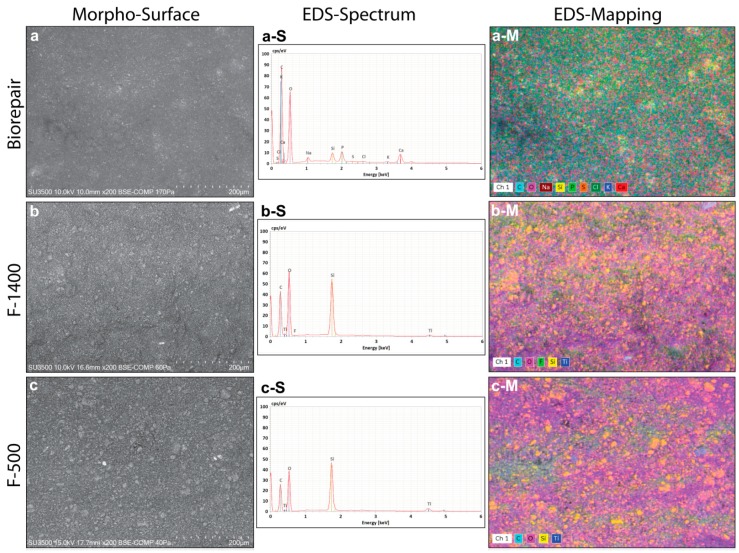
Morpho-chemical analysis of hydrated Biorepair, F1400, and F500 biomaterial toothpastes. (**a–c**) VP-SEM images illustrating the surface morphology. (**a-S**, **b-S** and **c-S**) EDS spectra probed on the corresponding biomaterials. (**a-M**, **b-M** and **c-M**) multi-elemental mapping image of major constituents.

The morpho-chemical analyses of the hydrated toothpastes are reported in Figure 2. The observations exhibited surface differences, where the Biorepair shows a smooth surface compared to F1400 and F500 fluorine toothpastes of the roughness surfaces (Figure 2a–c).

Interestingly, the EDS spectrum revealed also differences in the chemical compositions among toothpastes; indeed, inorganic material constituting an ensemble of C, O, Si, P, and Ca species essential for a proper enamel biomineralization at nanoscale synthesis was only detected for Biorepair (Figure 2a-S). On the other hand, F1400 of 1400 ppm contains only C, O, Si, F and Ti chemical elements similar to F500 expected for the fluorine element of 500 ppm, not technically detectable because of a low concentration (Figure 2b-S,c-S). Evidence of the micrometric roughness arrangement could be also seen by comparing the EDS mapping images, as displayed in Figure 2a-M, b-M, and c-M. In particular, micrometric aggregate of silicates (yellow colour) were present for both F1400 and F500 that cannot be a proper suitable dimension for diffusing inorganic nanomaterials through the enamel layer, but probably act only as a covering layer on the outer enamel. 

**Figure 3:**
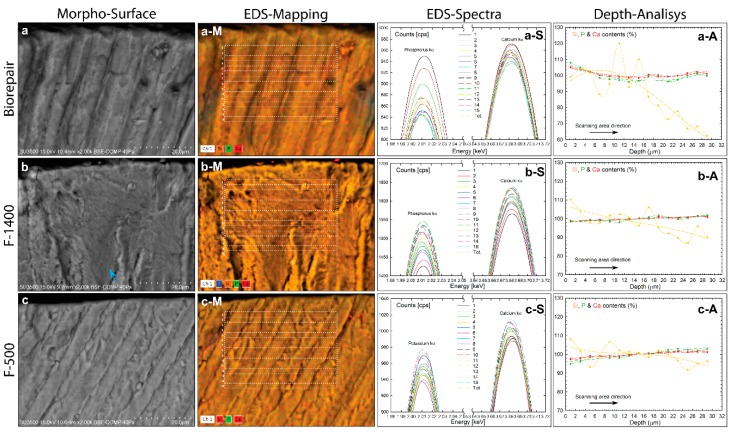
Morpho-chemical analysis of hydrated deciduous teeth brushed by Biorepair, F1400, and F500 biomaterial toothpastes. (**a**–**c**) VP-SEM images illustrating the surface morphology. (**a-S**, **b-S**, and **c-S**) EDS spectra probed on the corresponding biomaterials Figure 1d. (**a-M**, **b-M**, and **c-M**) multi- elemental mapping image of major constituents. (**a-A**, **b-A**, and **c-A**). Experimental plot showing atomic percentage distribution of Si, P, and Ca elements along the scanning area direction (dotted line) and data fitted by nonlinear regression for Biorepair and by linear regression for F1400 and F500 biomaterials (short dashed line).

To explore how commercial biomaterial toothpastes might evolve through the deciduous enamel layer after manual brushing, morpho-chemical imaging analyses of Biorepair, F1400, and F500 toothpastes were exploited to perform accurate quantitative measurements on the penetration depth and on the spatial chemical distribution (Figure 3).

Cross-sectioned surfaces of deciduous teeth show well-formed and aligned microrods interspaced by an enamel matrix. The data processing of the corresponding EDS spectra provided a quantitative elemental composition with Ca/P molar ratio of 1.84 ± 0.51% after Biorepair treatment and of 1.65 ± 0.45% for F500 (Figure 3a,c). The enamel deciduous of Figure 3b, exhibiting a layer of enamel matrix covering the crystalline microrods, showed a slight increase of the Ca/P ratio value of 1.75 ± 0.65% after F1400 treatment. The morpho-chemical analyses of the spatial distribution elements are shown in Figure 3a-M, b-M and c-M. Dual-EDS X-Ray mapping image exhibits a uniform distribution of the main chemical elements Si, P, and Ca on the entire mapped area. Similar to the proposed methodology analyses of the untreated teeth, additional information was extrapolated by analyzing the chemical spatial distribution across the enamel layer in terms of elemental content. It should be added that the cross-pattern position was not perfectly chosen on the enamel outermost border to avoid the highest chemical content rising up from the toothpaste influencing the fitting evaluation of the diffusion (slope contents). The experimental data of P and Ca scanning profile exhibits a best agreement using nonlinear regression fitting (Figure 3a-A, b-A and c-A) compared to the linear one of the untreated tooth (Figure 1h). The starting fitted curves have a slower decay profile with chemical contents up to 100% which confirms an absorption effect through the outer enamel surface due to the Biorepair treatment (Figure 3a-A). The ionic diffusion of P and Ca contents had a negative slope of −0.63 and −0.37 compared to the natural positive of the untreated tooth, confirming the absorption of the nanostructured Biorepair material at the outermost enamel surface, to decay in diffusion effect until a penetration depth of 16.10 ± 0.75 and 15.64 ± 0.68 μm on average, respectively. After that, the P and Ca contents along the scanning direction exhibited a positive slope similar to the untreated deciduous teeth (Table 1). Best nonlinear regression fitting was also applied to determine the ability of the inorganic silicate biomaterials to penetrate the enamel layer. The estimated delivery diffusion of the silicate’s Biorepair showed a start of lowest adsorption with a positive slope of 1.10 to reach a penetration depth of 6.91 ± 0.92 μm and decay quickly with a high negative slope of −2.08 (Figure 3a-A). Both F1440 and F500 were able to diffuse their microparticles of silicate through the enamel layer with starting Si contents of up to 100% of negative slope of −0.27 and −0.59, respectively. The calculated penetration depths were of 13.03 ± 0.69 and 14.78 ± 0.81 μm which further decreased the amounts of silicates diffused with slopes of −0.40 and −0.59 (Figure 3b-A,c-A).

## 4. Discussion

The investigated morphology, dimension and organization of the internal enamel layer of deciduous teeth shows a hierarchical assembly similar to the adult enamel crystals. The estimated dimension of the apatite rods is 4.63 μm smaller than the permanent enamel rod diameter of 6 μm, while the deciduous teeth showed a variation of rods from 4 to 7 μm [18,19]. On the other hand, the mean rod showed a close diameter to our results, varying from 3.22 μm to 3.47 μm for primary teeth and from 3.84 μm to 4.34 μm for permanent teeth [2]. These discrepancies can be related to the heterogeneous growth of the entire primary enamel layer in which the crystal elongation direction varies from the rod to the interrod [19], both growing differently in dimension especially in the aprismatic region. Therefore, the measured sizes of rod and interrod are strictly dependent on which area is estimated over the entire enamel layer. It should be added that the interesting interspacing rod matrix exerts its control through amorphous calcium phosphate precursor or imperfect oriented crystal attachment, as well-investigated by Beniash et al. [20,21]. To determine the amount of crystallized hydroxyapatite on the large micrometric area of enamel tooth, the typical evaluation of the enamel crystal growth by using the Ca/P molar ratio parameter exhibited a value of 1.54 barely lower compared to the permanent tooth enamel. The ratio of calcium to phosphate was estimated in the typical hydroxyapatite asf 1.67, while permanent dental enamel showed Ca/P molar ratio values ranging from 1.63 to 1.94 [22]. The authors asserted that the low enamel Ca/P was due to the interlayering combination of hydroxyapatite and octacalcium phosphate (Ca_8_(HPO_4_)_2_(PO_4_)_4_ 5H_2_O, 1.33 Ca/P) of early enamel crystallites. After Biorepair brushing, the increasing molar ratio at 1.84% would be favourable for growing the crystal habitat into deciduous enamel, as a biomimetic crystallization effect. This evidence of increasing calcium phosphate deposits qualitatively confirms the occurred penetration of the toothpaste within the outermost enamel layer. On the contrary, F1400 and F500 biomaterials showed a relative stable Ca/P (1.75 and 1.65) ratio due to the lack of calcium and phosphate contents in the toothpastes (Figure 2). High Ca/P molar ratio of Figure 3b compared to the untreated enamel teeth can be explained by the presence of the large interprismatic matrix visible in Figure 3b. The interprismatic layer could have accumulated more calcium phosphate deposits than the hydroxyapatite rods after treatment. It should be added that the slightly increased Ca/P molar ratio could be also influenced by silicate belonging to F1440 and F500 toothpastes. Biomaterial implant based on Si substitution in the calcium phosphate crystal structure has been shown to induce a slight increase in the molar ratio by increasing biological activity [23]. The estimated P and Ca contents locally into the smallest micrometric area, showed an increase in untreated deciduous teeth from the outer to the inner surface of the enamel layer. This experimental result validated the presence of the aprismatic region having low crystallinity organization at the outermost enamel surface in increasing progressively along the scanning area direction. Hence, the increase of crystallinity dimension in the long range order of the enamel hydroxyapatite in microrods is evident [24]. The role of biomimetic toothpastes in protecting and remineralizing the low crystallinity of the outer deciduous enamel showed a high penetration depth, estimated by the new method based on the EDS scanning area profile. After brushing treatments, Biorepair was capable of diffusing in depth its chemical aggregate constitutes of phosphate and calcium elements compared to the delivery diffusion in adult enamel at a depth penetration of 4.83 μm (P) and 2.65 μm (Ca) reported by Guentsh et al. [7]. These differences can be attributed to the different biomaterial product treatments since the enriched gelatin gel-film was a stationary application compared with our mechanical brushing treatments. This was also confirmed from the study on demineralized human dentin restored by new composite materials after complete immersion for 7 days, providing a highest penetration depth between 30 and 50 μm [8]. The proposed new method for estimating the penetration depth provides proper statistic acquisitions since it is based on the entire probed large area of 30 × 40 μm^2^. The presented diffusion of the silicate belonging to the biomimetic Biorepair showed substantial nonlinearity of the scanning profile which may be related to the clustering or accumulation effect of micrometric silicate within enamel during the diffusion processes (Figure 3a-A). P and Ca profiles of Biorepair studies have indeed the lowest oscillation due to their nanoscopic features of crystalline aggregate limiting the accumulation effect, as well-reported by Peetsch et al. [25]. On the other hand, the phosphate and calcium depth curves of the Figure 3b-A,c-A had a positive slope similar to the untreated teeth since the F1440 and F500 toothpastes did not contain P and Ca elements, as shown in the EDS spectrum of Figure 2b-S,c-S. Whereas, the g silicon profile belonging to the fluorine toothpastes had still nonlinearity behavior, the starting uptake into the outermost enamel layer was different, showing high chemical contents up to 100%. This difference can be related to the high amount of silicate in the fabricated fluorine biomaterials, as shown in the EDS-spectra of Figure 2b-S,c-S. The high amount of silicon in the toothpaste has been considered to promote the formation of apatite crystasl, but only in a supersaturated solution of Ca^2+^ and PO_4_^3−^ ions and further the calcium-silicate biomaterial has the ability to release mineral ions [8,26]. Furthermore, silicate compounds have also been proposed to buffer environmental acids due to their high thermal/hydrothermal stability [27]. By comparing the silicate diffusions of the three different biomimetic toothpastes, let us assert that the behavior diffusion profile of Biorepair silicate can be attributed to the multidimensional formation of silicate particles at either micrometric or nanometric scale [25]. On the other hand, the different nonlinearity behavior of the silicate diffusion is conditioned by the formation of predominant microaggregates in high percentage contained in F1500 and F400 biomaterials observable in Figure 2b-M,c-M.

The chemical and dimensional constituents of Biorepair have been shown to better diffuse in the enamel layer. Consequently, Biorepair may potentially contribute to remineralize the early stage or the loss of the enamel mineral in deciduous teeth since these biomaterials have a great similarity to the composition of natural teeth. Although, phosphate and calcium are not present in F1400 and F500, the high amount of silicate detected may confer some protective action to these commercial toothpastes from the stimulating external agent in order to decrease the porosity and structural weakness of the tooth. However, it is notable that the action of the biomimetic toothpastes by mechanical brushing is underestimated for their abrasive processes especially for the detected microaggregate of silicate of the F1500 and F400 toothpastes, acting as a corrosive process [28]. Low abrasion due to the chemical aggregates of calcium carbonate contained in Biorepair have been shown to be due to the softness of the biomimetic materials relative to the other abrasive dental products. Therefore, an ideal biomaterial toothpaste should contain a chemical ensemble of calcium, phosphates, silicon, oxygen, etc. However, the chemical composition of a toothpaste is not only the unique feature of the remineralization process since the dimensional aggregation of the inorganic biomaterials play a primary role in diffusion transport of remineralization ions. The observed microscopic particles of F1400 and F500 have not a proper dimension to diffuse through to the enamel surface for growing and nucleating crystals; while nanoscopic biomaterial particles of the Biorepair have been evaluated as an essential dimension to be adsorbed by the enamel surface for repairing enamel lesion and for remineralization treatments [25,29].

These preliminary results suggest promising remineralization obtained with toothpastes containing nanocrystals of hydroxyapatite. This aspect should be studied in depth, not only to further support the outcomes supplied by the present study, but also to provide a reliable alternative to toothpaste containing fluorine that, although having valuable protective action, may cause fluorosis as a result of excessive assumption [30].

## 5. Conclusions

The deciduous teeth treated by three different toothpaste biomaterials (Biorepair, F1400, and F500) penetrating into the enamel layer were thoroughly investigated in all morpho-chemical aspects. The new approach proposed here, provides direct experimental evidence on the delivery diffusion performances of commercially available toothpastes. By tailoring synthetic graininess in biofluid media, the toothpastes were to be capable of reaching building blocks of both enamel matrix and apatitic mineral phase by passing through the outermost enamel surface. To get an insight into the diffusion behaviour of the biomimetic toothpastes, electron microscopy imaging techniques supported by simply processing data of EDS imaging analyses were exploited. We were able to determine several morpho-chemical features: (*i*) the microscopy investigations showed both the ability of the deciduous enamel to be assembled into a 3D-network and its evolution in terms of chemical distribution contents (Figure 1); (*ii*) the processing data of EDS imaging analyses evidenced the delivery diffusion behaviour of the silicate, phosphate, and calcium inorganic species assembled in a biomaterials toothpastes. (Figure 2 and Figure 3); (*iii*) for expanding the focus of research, the diffusion performances of the investigated biomaterials were shown to be dependent of the multidimensional granularity and chemical contents of the biomimetic particles either at micrometric or nanometric scale.

Finally, these achievements provide concrete information useful not only for understanding the diffusion mechanisms of nanostructured microaggregate, but also for tailoring specific chemical and dimensional dental materials as a viable alternative to synthetic fluoride-containing toothpastes. These findings will be useful in the current challenging research for developing a controlled toothpaste in biofluid media able to modulate protective and biomineralization properties and to be used in different applications of regeneration.

## Figures and Tables

**Table 1:** Mean diffusion coefficient slope and penetration depth extrapolated by linear and non-linear regression of the atomic content curves.

Toothpaste/Elements	Slope			Penetration Depth (μm)		
	Si	P	Ca	Si	P	Ca
**Free**	―	0.30 ± 0.03	0.30 ± 0.06	―	―	―
**Biorepair**	1.10 ± 0.05 & −2.08 ± 0.02	−0.63 ± 0.01 & 0.42 ± 0.01	−0.37 ± 0.01 & 0.29 ± 0.01	6.91 ± 0.92	16.10 ± 0.75	15.64 ± 0.68
**F1400**	−0.27 ± 0.01 & −0.40 ± 0.01	0.26 ± 0.03	0.14 ± 0.01	13.03 ± 0.69	―	―
**F500**	−0.59 ± 0.01 & −0.59 ± 0.01	0.11 ± 0.02	0.09 ± 0.01	14.78 ± 0.81	―	―

*p* < 0.01 statistical significant difference between experimental and theoretical data fitting.
